# Prevalence, Incidence and Predictors of Anal HPV Infection and HPV-Related Squamous Intraepithelial Lesions in a Cohort of People Living with HIV

**DOI:** 10.3390/diagnostics15020198

**Published:** 2025-01-16

**Authors:** Margherita Sambo, Alessandra Bailoni, Federico Mariani, Massimo Granai, Natale Calomino, Virginia Mancini, Anna D’Antiga, Francesca Montagnani, Mario Tumbarello, Stefano Lazzi, Franco Roviello, Massimiliano Fabbiani

**Affiliations:** 1 Department of Medical Biotechnologies, University of Siena, Viale Mario Bracci 16, 53100 Siena, Italy; margherita.sambo@unisi.it (M.S.); alessandra.bailoni@unifi.it (A.B.); anna.dantiga@student.unisi.it (A.D.); francesca.montagnani@unisi.it (F.M.); mario.tumbarello@unisi.it (M.T.); 2 Infectious and Tropical Diseases Unit, Azienda Ospedaliero-Universitaria Senese, 53100 Siena, Italy; 3 Department of Medicine, Surgery and Neurosciences, Unit of General Surgery and Surgical Oncology, University of Siena, 53100 Siena, Italy; fed-mar@live.it (F.M.); natale.calomino@unisi.it (N.C.); franco.roviello@unisi.it (F.R.); 4 Institute of Pathology, Department of Medical Biotechnology, Azienda Ospedaliero-Universitaria Senese, 53100 Siena, Italy; massimo.granai@unisi.it (M.G.); virginia.mancini@unisi.it (V.M.); lazzi2@unisi.it (S.L.)

**Keywords:** HIV, human papilloma virus (HPV), PLWH, screening, squamous intraepithelial lesion, anal squamous cell carcinoma, HPV vaccination

## Abstract

**Background:** Anal HPV infection can cause squamous intraepithelial lesions (SILs), which are precursors of anal squamous cell carcinoma (SCC). The early detection of HPV infections and improvement of effective screening programmes are, therefore, essential to prevent progression from pre-cancerous lesions to SCC, especially in people living with HIV (PLWH), who represent a population at higher risk of HPV infection and associated lesions. Among prevention strategies, HPV vaccination is relevant too, but its efficacy in persons already infected by HPV is still debated. **Methods**: This is a retrospective single-center study on a cohort of PLWH who performed longitudinal screening for anal dysplasia and HPV infection. The screening included cytological and molecular analyses. **Results**: A total of 110 PLWH performed at least one anal HPV screening, with an overall prevalence of HPV infection of 86.4% [23.6% low risk (LR)-HPV and 62.7% high risk (HR)-HPV genotypes]. Abnormal cytology was demonstrated in 39.1% of subjects, of whom ASCUS 6.4%, LSIL 30.9% and HSIL 1.8%. In total, 80 patients (72.7%) had an available longitudinal screening. No patient developed SCC during follow-up. However, a high incidence of new cytological abnormalities and new HPV infections was observed. On the other side, clearance of some HPV genotypes was also frequent, confirming that HPV infection is a dynamic process. A CD4 cell count > 500/mmc was an independent predictor of HPV clearance. HPV vaccination was performed on 30.9% of patients. A trend toward an increased clearance of HPV genotypes included in 9-valent vaccine was observed in vaccinated patients (40.6% versus 30.8% in unvaccinated, *p* = 0.079). **Conclusions**: A high prevalence of HPV infection and SILs was observed in our cohort of PLWH. A high incidence of new HPV infections and HPV-associated lesions was also observed in the longitudinal cohort, highlighting the need of strengthening immunization programs and continuous screening for anal HPV infection. Whether HPV vaccination may be efficacious in patients already infected by HPV remains to be determined.

## 1. Introduction

Anal Human Papilloma Virus (HPV) infection can cause squamous intraepithelial lesions (SIL), which are precursors of anal squamous cell carcinoma (SCC). People living with HIV (PLWH) are a population at higher risk of HPV infection and HPV-associated lesions [1,2,3], and therefore deserve particular attention in terms of screening and prevention strategies.

In order to prevent the evolution of HPV infection and HPV-related lesions, it is essential to detect infected patients and to implement effective screening programs to early identify the onset of pre-cancerous lesions and their potential progression toward SCC [4,5]. However, relatively little is known about the long-term natural history of anal SILs, which is believed to be a dynamic process, with lesions arising and regressing over time [6]. Nevertheless, the benefits of screening and monitoring lesions over time are supported by several reasons [7]: (i) the high incidence of anal cancer in the populations for which screening is offered [8]; (ii) the availability of screening methods that can effectively diagnose high-grade SILs (HSILs) [9]; (iii) the stage at which anal cancer is detected has a substantial impact on survival rates, so early detection is crucial [10]; (iv) the significant morbidity and mortality associated with anal cancer can be largely prevented by early screening [10]. Therefore, it is crucial to analyze the dynamics of anal HPV infection and anal cytology over time.

The diagnostic screening algorithm for anal lesions rely on digital anorectal inspection, HPV molecular testing, cytology and high-resolution anoscopy [8,11,12]. High-resolution anoscopy (gold standard) is generally reserved for those patients who first show altered results on cytology, as this technique is expensive and invasive [13].

HPV vaccination also plays an important role for the prevention of HPV-related neoplastic lesions in PLWH and, in recent years, it is offered to all prepubescent girls and boys. In the United States, the ACIP recommends HPV vaccination for all children aged 11 to 12 years, and for everyone up to 26 years if they were not adequately vaccinated when younger. HPV vaccination may also be considered for adults aged 27 to 45 years, based on a case-by-case basis considering additional risk factors (e.g., HIV infection) [14]. Several data demonstrated that HPV vaccination is safe in the presence of HIV [15,16,17,18,19,20,21], even if some studies show a weaker and more transient immune response when compared to the general population [19,22,23]. However, knowledge about this topic is still scanty and the role and persistence of vaccine protection in PLWH are still widely debated [24,25].

Few studies previously investigated the efficacy of HPV vaccination in formerly HPV-infected people, especially if infected by HIV [26,27,28,29]. Thus, the clinical value of vaccination is still to be determined in this setting. Consequently, it is crucial to examine the effects of HPV vaccination in PLWH already infected by HPV.

The aim of this study was to analyze the relationship between HIV infection and HPV infection over time, taking into account the incidence of:

- Occurrence of new infections: in particular any new HPV infection, new Low-Risk HPV (LR-HPV) infection, new High-Risk HPV (HR-HPV) infection and the presence of new infections by HPV genotypes included in the 9-valent vaccine.
- Clearance of any HPV genotype, clearance of LR-HPV and HR-HPV genotypes and clearance of HPV genotypes included in the vaccine formulation.
- Occurrence of new HPV lesions (Low Grade SIL, HSIL) and their potential progression over time.

Moreover, a secondary study aim was to investigate if HPV vaccine may interact with the occurrence of new infections and/or clearance of previous infections, as well as to evaluate its role in reducing the progression of SILs and the occurrence of new HPV-related lesions.

## 2. Materials and Methods

This is a retrospective single-center study including PLWH in regular follow-up at the Infectious and Tropical Diseases Clinic of the Azienda Ospedaliera Universitaria Senese (AOUS) which performed longitudinal screening for anal dysplasia and HPV infection for routine clinical practice from January 2018 to March 2024. Age < 18 years was the only exclusion criterion. According to current guidelines [30] and to our internal protocol, at our center anal HPV screening is proposed annually to all men who have sex with men (MSM) and all subjects with HPV infection or HPV-associated dysplasia at any site. The screening includes cytological analysis and molecular analysis.

Cytological analysis was performed by anal brushing (anal PAP test) and cytological abnormalities were defined according to the Bethesda classification into: no cytological abnormalities, atypical squamous cells of uncertain significance (ASC-US), low-grade squamous intraepithelial lesions (LSIL), high-grade squamous intraepithelial lesions (HSIL) and lesions that could not be evaluated (i.e., less than 2000 to 3000 nucleated squamous cells) [31].

Molecular testing was also performed to recognize multiple HPV genotypes by multiplex PCR (Genomic DNA FFPE ONE-STEP kit), distinguishing between high risk (HR-HPV) and low risk (LR-HPV) genotypes [High risk (HR): 16, 18, 31, 33, 35, 39, 45, 51, 52, 56, 58, 59, 66, 68; Low risk (LR): 6, 11, 40, 42, 43, 44, 54, 61, 70, 72, 81, 84, 89, 90, 102].

Patients were followed from baseline (BL, time of first screening for anal HPV infection) to the last available outpatient visit. Clinical and laboratory variables were obtained from the medical record review: age, sex, risk factors for HIV infection (heterosexual, homosexual/bisexual, unspecified, other/unknown), country of origin, HPV vaccination status. Viroimmunological, therapeutical and biochemical parameters were also considered, as well as comorbidities and other sexually transmitted infections.

From these data, a database was created from which statistical analyses were then performed. Data collection was approved by the local ethic committee and informed consent was obtained from all patients before participation. The study was carried out in accordance with the ethical principles of the Declaration of Helsinki and with the Good Clinical Practice guidelines of the International Conference on Harmonization.

All patients who had at least one anal cytology screening (110 patients) were included in the study and contributed to the descriptive cross-sectional population analysis. The estimated and calculated incidence analyses were performed on subjects who had at least one post-BL follow-up (80 out of 110, longitudinal cohort).

The following outcomes were evaluated for incidence analyses: (i) any new cytological abnormality, defined as the appearance of cytological abnormalities in those with normal BL anal pap test (ii) new HSIL, defined as occurrence of new HSIL lesions in patients without previous HSIL evidence (iii) worsening cytological abnormalities, defined as worsening of cytological stage (from ASCUS to LSIL to HSIL to anal SCC) (iv) clearance of any HPV, defined as any confirmed (at least two consecutive tests) clearance of a HPV genotype during follow-up (v) clearance of LR-HPV, defined as any confirmed (at least two consecutive tests) clearance of a LR-HPV genotype during follow-up (vi) clearance of HR-HPV, defined as any confirmed (at least two consecutive tests) clearance of a HR-HPV genotype during follow-up (vii) any new HPV infection, defined as the detection of any new HPV genotype not previously detected (viii) new LR-HPV infection, defined as the detection of any new LR-HPV genotype not previously detected (ix) new HR-HPV infection, defined as the detection of any new HR-HPV genotype not previously detected. A sub-analysis evaluating new infections or clearance of any HPV genotypes included in the 9-valent HPV vaccine was also performed.

Descriptive statistics (number, proportion, median, interquartile range, 95% confidence interval) were used to describe the baseline characteristics of the patients. Categorical variables were compared between groups using the χ2 test or Fisher’s exact test, as appropriate. Continuous variables were compared using the non-parametric Mann-Whitney U-test. Only values of *p* < 0.05 were considered significant. To evaluate the occurrence of the different outcomes over time, their incidence was calculated as 100-person year of follow-up (PYFU) with a 95% confidence interval (CI). Kaplan–Meier curves were then used to calculate the estimated incidence at different time points, using log rank test to compare subgroups. We also explored predictors of a new HPV infection or clearance of any HPV genotype by Cox regression analysis; variables associated with the outcome at univariate analysis were then evaluated in a multivariate model to confirm independent associations.

All analyses were performed using SPSS software v.25.0 (SPSS Inc., Chicago, IL, USA).

## 3. Results

### 3.1. Population Characteristics

A total of 110 PLWH aged between 43 and 56 years (median age 49) were included, of whom 85.5% were males. Population characteristics are reported in Table 1. The population had the following risk factors for HIV infection: 14.5% were heterosexual patients, while over 54.5% were men who have sex with men (MSM), 0.9% injecting drug users (IDU) and 30% had unknown/other risk factors.

Among this population, 34 patients (30.9%) were vaccinated for HPV, of which only 10 received the vaccine before BL (9.1%). Six of the 34 vaccinated subjects (17.6%) did not complete the full vaccination schedule (3 and 3 patients performed only 1 and 2 doses, respectively).

On average, the median number of years since HIV diagnosis was 8.5. Twenty (18.2%) patients had a history of AIDS events [overall 29 events, the most frequent were: 6 (20.7%) *Pneumocystis jirovecii* pneumonia, 6 (20.7%) Kaposi Sarcoma, 4 (13.8%) oesophageal candidiasis, 3 (10.3%) non Hodgkin lymphomas, 3 (10.3%) wasting syndromes]. At BL, 3 patients (2.7%) were positive for HbsAg, 36 (32.7%) were positive for HbcAb, 5 (4.5%) had a previous HCV infection and 32 (29.1%) had a history of syphilis. Regarding HIV infection, 84.5% of patients had HIV-RNA < 50 copies/mL and a median CD4 lymphocyte count of 643 cells/mmc, with a median CD4/CD8 ratio of 0.90.

### 3.2. HPV Infection and Cytological Abnormalities

The presence of HPV infection and cytological abnormalities at baseline and their evolution over follow-up are detailed in Table 2. At first screening, anal HPV infection was observed in 95 (86.4%) patients, including 26 LR-HPV genotype infections (23.6%) and 69 HR-HPV genotype infections (62.7%). The presence of abnormal cytology was demonstrated in 43 subjects (39.1%), of whom ASCUS *n* = 7, 6.4%; LSIL *n* = 34, 30.9%; HSIL *n* = 2, 1.8%. A total of 33 (30%) patients had lesions that could not be evaluated according to Bethesda classification; of these, only 7 (21.2%) subjects did not show HPV infection, while 7 (21.2%) and 19 (57.6%) individuals demonstrated LR-HPV and HR-HPV, respectively.

A total of 80 (72.7%) PLWH repeated screening during follow-up (longitudinal cohort). The characteristics of patients in the longitudinal cohort were similar to those who did not perform repeated HPV testing, with the exception of a longer history of HIV infection (median 14 versus 8 years, *p* = 0.011) and a longer exposure to ART (median 10 versus 5 years, *p* = 0.021) in the former group. In the longitudinal cohort, 28 (35%) received HPV vaccination.

During a median follow-up of 35.3 months (interquartile range, IQR 20.4–49.0) with a median of 4 (IQR 2.3–5) screenings for each patient, no cases of SCC were observed. The presence of any new cytological atypia during follow-up was found in 57 patients (71.3%), the presence of HSIL in 6 patients (7.5%), the progression of cytological lesions in 6 patients (7.5%). Evaluating HPV infection during the follow-up period, 58 cases (72.5%) of new HPV infection occurred and clearance of any HPV genotype in 48 subjects (60.0%) was recorded.

### 3.3. HPV Vaccination

Characteristics of HPV-vaccinated (*n* = 34) or unvaccinated (*n* = 76) patients are reported in Table 3. The median age of the vaccinated population was significantly lower (47 years) than that of the unvaccinated (50 years) (*p* = 0.014). Moreover, vaccinated subjects had a shorter time from HIV diagnosis [median 3.0 years (IQR 2.0–13.5) versus 11 years (IQR 5.0–17.0), *p* = 0.001] and from antiretroviral therapy initiation [median 3.0 years (IQR 1.0–10.0) versus 8.0 years (IQR 4.0–14.0), *p* = 0.001] than unvaccinated individuals. Vaccinated and unvaccinated patients did not show significant differences in other main characteristics (e.g., risk factor, coinfections, viral load, CD4 count, antiretroviral regimen).

The presence of HPV infection and cytological abnormalities at baseline and their evolution over follow-up in vaccinated and unvaccinated patients are detailed in Table 4.

At BL, the proportion of HPV infection in vaccinated patients was 82.4% (of which 26.5% with LR-HPV infection and 55.9% with HR-HPV infection) compared to 88.2% (of which 22.4% present LR-HPV infection and 65.8% present HR-HPV infection) in unvaccinated (*p* = 0.604).

At first screening, no cytological abnormalities were seen in 32.4% of vaccinated and 30.3% of unvaccinated patients, respectively (*p* = 0.348). Cytological abnormalities in vaccinated versus unvaccinated subjects were: ASC-US 0% versus 9.2%, LSILs 35.3% versus 28.9%, HSILs 0% versus 2.6%; moreover, there were some lesions that could not be evaluated (32.4% in vaccinated and 28.9% in unvaccinated patients).

The percentage of patients with an available follow-up after the first screening was 82.4% in the vaccinated population and 68.4% in the non-vaccinated population (*p* = 0.199). The duration of follow-up was significantly different in the two groups (*p* = 0.036), with an average of 36.9 months in the vaccinated and 20 months in the unvaccinated.

The vaccinated population who was followed over the time showed an occurrence of any new cytological abnormalities in 58.8% of cases versus 59.2% in unvaccinated. Of these new lesions, the occurrence of HSIL was 5.9% in the vaccinated and 5.3% in the unvaccinated groups. The rate of worsening of cytological lesions was 8.8% in the vaccinated and 3.9% in the unvaccinated (*p* = 0.371).

Regarding HPV infection during follow-up, there was a significantly higher rate of any new HPV infections (70.6%) in the vaccinated group of patients compared to the unvaccinated (44.7%) (*p* = 0.021). Of all acquired infections, those from genotypes included in the 9-valent vaccine were 13/21 (61.9%) in vaccinated and 26/74 (35.1%) in unvaccinated patients (*p* = 0.051).

During follow-up, the clearance of infection occurred in 52.9% and 39.5% in the vaccinated and unvaccinated population, respectively. In particular, the clearance of genotypes contained in the 9-valent vaccine was 61.9% and 35.1% in the vaccinated and non-vaccinated population, respectively (*p* = 0.051).

### 3.4. Incidence of HPV Infection/Clearance and Evolution of Cytological Abnormalities over Time

We further analyzed the incidence of HPV infection/clearance and the evolution of cytological abnormalities over time in patients with an available follow-up. The overall number of patients in regular follow-up was 80, of which 28 vaccinated subjects and 52 unvaccinated subjects. Of the 43 subjects with abnormal cytology at baseline, 35 (81.4%) were included in the longitudinal cohort. The median follow-up was 35.3 months (95% CI 24.8–45.8).

During follow-up, the incidence of the different outcomes was as follow: (i) any new cytological abnormality 20 per 100 PYFU (95% CI 8.3–31.7), (ii) new HSIL 2.4 per 100 PYFU (95% CI −0.4–5.2), (iii) worsening cytological abnormalities 2.3 per 100 PYFU (95% CI −0.5–5.1), (iv) clearance of any HPV 24.9 per 100 PYFU (95% CI 16.8–33.0), (v) clearance of LR-HPV 15.1 per 100 PYFU (95% CI 8.4–21.8), (vi) clearance of HR-HPV 13.3 per 100 PYFU (95% CI 7.0–19.6), (vii) any new HPV infection 38.2 per 100 PYFU (95% CI 29.1–47.2), (viii) new LR-HPV infection 9.6 per 100 PYFU (95% CI 4.1–15.1), (ix) new HR-HPV infection 28.6 per 100 PYFU (95% CI 20.2–37.0).

Kaplan–Meier analysis was used to estimate incidence of the different outcomes at various time points (see Supplementary Figure S1). At 24 months, incidence was as follow in the overall population: (i) any new cytological abnormalities 33.1% (95% CI 18–48.2), (ii) new HSIL 3.1% (95% CI −0.4–6.6), (iii) worsening cytological abnormalities 2.7% (95% CI −0.8–6.2), (iv) clearance of any HPV 39.5% (95% CI 28.1–50.9), (v) clearance of LR-HPV 23.9% (95% CI 13.5–34.3), (vi) clearance of HR-HPV 20.9% (95% CI 11.5–30.3), (vii) any new HPV infections 54.7% (95% CI 42.7–66.7), (viii) new LR-HPV infection 20.6% (95% CI 10.8–30.4), (ix) new HR-HPV infection 38.3 (95% CI 26.5/50.1).

**Figure 1:**
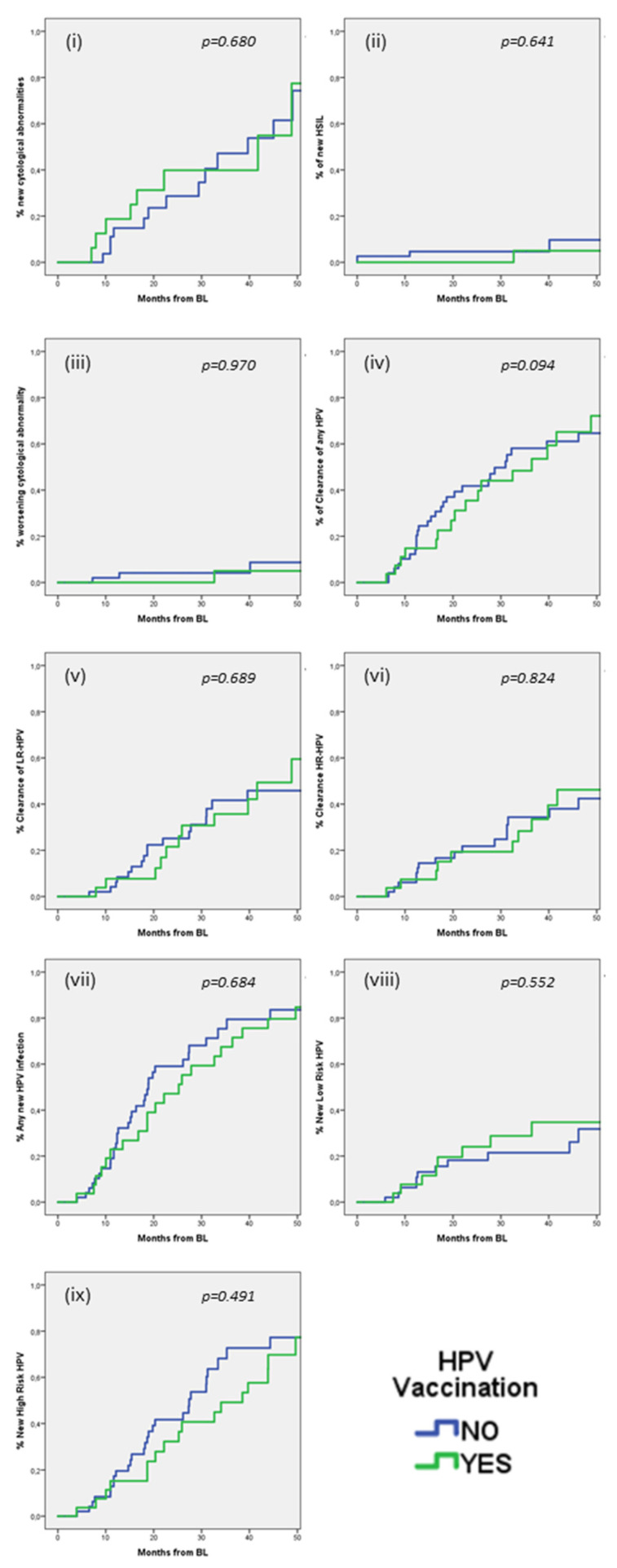
Kaplan–Meier estimates of the Incidence of HPV infection/clearance and evolution of cytological abnormalities over time in vaccinated and unvaccinated patients. Notes: (**i**) any new cytological abnormality; (**ii**) new HSIL; (**iii**) worsening cytological abnormalities; (**iv**) clearance of any HPV; (**v**) clearance of LR-HPV; (**vi**) clearance of HR-HPV; (**vii**) any new HPV infection; (**viii**) new LR-HPV infection; (**ix**) new HR-HPV infection.

We also explored if HPV vaccination could influence these outcomes (Figure 1). No significant differences were observed in any of the outcomes (i–ix) between the group of HPV vaccinated and the group of HPV unvaccinated PLWH. At 24 months, the estimated incidences in vaccinated versus unvaccinated patients were: (i) any new cytological abnormalities 39.8% (95% CI 14.5–65.1) versus 28.7% (95% CI 10.5–46.9) (*p* = 0.680), (ii) new HSIL 0% versus 4.7% (95% CI −0.6–10.0) (*p* = 0.641), (iii) worsening cytological abnormalities 0% versus 4.1% (95% CI −1.6–9.8) (*p* = 0.970), (iv) clearance of any HPV 35.5% (95% CI 16.7–54.3) versus 41.8% (95% CI 27.7–55.9) (*p* = 0.094), (v) clearance of LR-HPV 21.5% (95% CI 4.6–38.4) versus 25.1% (95% CI 12.2–38.0) (*p* = 0.689), (vi) clearance of HR-HPV 19.4% (95% CI 4.1–34.7) versus 21.8% (95% CI 9.6–34.0) (*p* = 0.824), (vii) any new HPV infections 47.1% (95% CI 27.7–66.5) versus 59.1% (95% CI 44.2–74.0) (*p* = 0.684), (viii) new LR-HPV infection 24.0% (95% CI 7.1–40.9) versus 18.2% (95% CI 6.6–29.8) (*p* = 0.552), (ix) new HR-HPV infection 32.2% (95% CI 13.8–50.6) versus 41.7% (95% CI 26.8–56.6) (*p* = 0.491).

**Figure 2:**
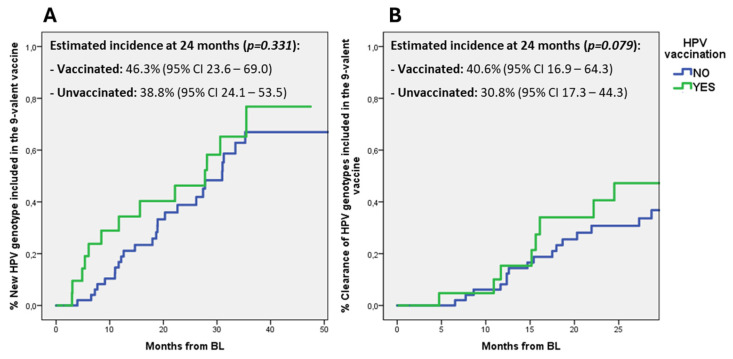
Kaplan–Meier estimates of the incidence of new infections or clearance of HPV genotypes included in the 9-valent HPV vaccine in vaccinated and unvaccinated patients. Notes: (**A**) new infection by HPV genotypes included in 9-valent vaccine; (**B**) clearance of HPV genotypes included in 9-valent vaccine.

Furthermore, we also explored if HPV vaccination could influence the incidence of new infections or clearance of HPV genotypes included in the 9-valent HPV vaccine. While no differences were observed between vaccinated and unvaccinated patients in the incidence of new infections by HPV genotypes included in the 9-valent vaccine, vaccinated subjects showed a trend toward an increased clearance of vaccinal HPV genotypes when compared to unvaccinated: at 24 months the estimated incidence was 40.6% (95% CI 16.9–64.3) versus 30.8% (95% CI 17.3–44.3), respectively (*p* = 0.079) (Figure 2).

### 3.5. Predictors of HPV Infection and Clearance over Time

We also explored whether demographic, HPV-related, or HIV-related variables could be associated with the occurrence of a new HPV infection or clearance of any HPV genotype, using Cox regression analysis (Table 5).

In univariate analysis, prior AIDS-related events were associated with a lower probability of new HPV infections (hazard ratio, HR 0.40, 95% CI 0.17–0.95, *p* = 0.037), while a CD4 count > 500 cells/mm^3^ was associated with a higher probability (HR 1.89, 95% CI 1.03–3.47, *p* = 0.041). However, statistical significance was not confirmed in multivariate analysis [AIDS: adjusted HR (aHR) 0.48, 95% CI 0.20–1.16, *p* = 0.103; CD4 > 500 cells/mmc: aHR 1.58, 95% CI 0.84–2.97, *p* = 0.155).

Exploring predictors of HPV clearance, in univariate analysis subjects with HR-HPV showed a lower probability (HR 0.50, 95% CI 0.28–0.91, *p* = 0.023), while those with CD4 count > 500 cells/mmc had a higher probability (HR 2.62, 95% CI 1.24–5.54, *p* = 0.011) of any HPV genotype clearance. However, in the multivariate analysis only CD4 cells > 500/mmc remained an independent predictor of HPV clearance (aHR 2.44, 95% CI 1.15–5.18, *p* = 0.021), while infections with HR-HPV genotypes showed only a nearly significant trend towards a reduced probability of clearance (aHR 0.56, 95% CI 0.31–1.01, *p* = 0.054).

## 4. Discussion

In our study, we evaluated the prevalence and the evolution over time of anal HPV infection and HPV-associated cytological abnormalities in a population of PLWH. At the first screening, we observed a high prevalence (86.4%) of anal HPV infection. This high prevalence is similar to that reported in other cohorts of PLWH [12,32,33,34], which are a population at increased risk of HPV infection and its associated pre-cancerous/cancerous lesions [35]. Moreover, our cohort was predominantly constituted by MSM, which are a group at increased risk of anal HPV infection, and this could have contributed to the high prevalence of HPV infection [36].

Since most HPV infections were sustained by HR-HPV genotypes (62.7%), this could translate in a higher risk of developing cytological abnormalities. Indeed, at the first screening, cytological abnormalities were quite common in our cohort, being recognized in 39.1% of patients. Most of them were classified as LSIL (30.9%), with only 1.8% of HSIL and no cases of SCC. Since the evolution of SILs toward overt cancerous lesions is increased in PLWH, the high prevalence of SIL in our cohort highlights the importance of a longitudinal screening program in PLWH to early diagnose anal HPV infection and pre-cancerous lesions to prevent anal SCC [4,11].

Despite repeated screenings being proposed to all subjects in our cohort, only 72.7% consented to re-testing and had an available follow-up. This highlights the importance of strengthening counseling to all PLWH at risk for HPV infection, to improve adherence to screening procedures. Moreover, several structural and organizational adjustments should be considered at a local level to link screening procedures for HPV to the routine clinical care of PLWH, thus making screening compliance easier.

During follow-up, no cases of anal SCC were observed in our cohort. However, in the longitudinal cohort, the proportion of patients with cytological abnormalities increased to 71.3%, with 7.5% of patients showing a progression of cytological abnormalities over time and 7.5% developing HSIL. The incidence of any new cytological abnormalities was 20 per 100 PYFU, with an incidence of HSIL and of worsening cytological abnormalities of 1.6 and 2.3 per 100 PYFU, respectively. These data confirm that anal lesions can evolve over time, thus further enhancing the relevance of implementing longitudinal screening for SCC prevention. It will be relevant to evaluate if a further progression of cytological abnormalities and eventually the appearance of cases of anal SCC will be observed by prolonging follow-up.

In our longitudinal cohort, we also explored the dynamics of HPV infection over time by evaluating the appearance/clearance of HPV genotypes and we estimated incidence of various outcomes. In general, a dynamic process was observed with appearance and clearance over time of different genotypes also in the same patient. Overall, 60% of patients showed the clearance of at least one HPV genotype, with an incidence of 24.9 per 100 PYFU. The proportion of clearance was similar for HR-HPV and LR-HPV (38.8% and 37.5%, respectively). On the other side, appearance of new HPV genotypes was also observed in a substantial proportion of patients, since during follow-up 72.5% showed new genotypes not detected at baseline, with an incidence of 38.2 per 100 PYFU. This was more common for HR-HPV than for LR-HPV (61.3% versus 21.3% of subjects, respectively). These data can have several explanations. HPV persistence in tissues is a dynamic process that is influenced by a balance between HPV replication and immune system activity [37,38]. Indeed, clearance of HPV genotypes can be the result of an adequate immune pression on the virus. However, also inadequate sampling could have played a role. On the other side, the appearance of new HPV genotypes could be the result of new HPV infections or of HPV reactivation due to an impaired immune system.

The acquisition of HPV and its dynamics over time can be influenced by HPV vaccination, the effectiveness of which is widely documented also in PLWH for primary prevention [20]. In our population, only 9.1% of patients had received HPV vaccine before baseline. The low proportion of patients previously vaccinated against HPV can partly be related to the median age (49 years) of our population, mainly constituted by subjects born when universal HPV vaccination in prepubertal children had not yet been introduced. Indeed, median age of vaccinated patients was lower than that of unvaccinated (47 versus 50 years). The proportion of vaccinated subjects increased to 30.9% after first screening, despite the efficacy of HPV vaccine for preventing anal SCC in adult PLWH who already acquired HPV is controversial [39].

We also explored if HPV vaccination could influence HPV acquisition/clearance and the evolution of cytological abnormalities. No significant differences were observed between the group of HPV vaccinated and the group of HPV unvaccinated patients in the incidence of any new cytological abnormality, clearance or acquisition of any HPV. However, 9-valent vaccinated subjects showed a trend toward an increased clearance of HPV vaccinal genotypes (40.6% versus 30.8% when compared to unvaccinated, *p* = 0.079). Although not reaching statistical significance partly due to the small sample size, this observation raises questions whether HPV vaccination may contribute to enhance the immunological response against genotypes it contains, potentially leading to HPV clearance in patients who had previously acquired the infection.

In our population, we could not demonstrate any independent predictors of infections with new HPV genotypes. However, a CD4 count > 500 cells/mmc was independently associated with a higher likelihood of HPV clearance, suggesting that a compromised immune system may play a relevant role in HPV persistence. We also observed that HR-HPV genotypes were less likely to be cleared over time than LR-HPV, thus highlighting the importance of strengthening prevention measures (e.g., vaccination) to prevent HR-HPV infections, which are associated with an increased risk of cytological abnormalities and progression to SCC. Some limitations should be recognized when interpreting the results of our study. Its retrospective and observational design could have introduced potential biases, since patients undergoing screening procedures could have been selected based on unaccounted variables. However, our protocol for PLWH recommends HPV screening in all MSM and other groups at high risk for HPV infection and its related cytological abnormalities, therefore, screening should have been proposed to all eligible patients. Not all patients were adherent to longitudinal screening, the duration of follow-up was limited (median nearly 3 years) and few subjects performed the suggested HPV vaccination, thus reducing sample size and consequently statistical power of the analyses. Based on this, it is not possible to draw definitive conclusions on whether the vaccine is effective or not in preventing new infections and/or increasing the resolution of previous infections. Moreover, at baseline some patients had lesions that could not be evaluated according to Bethesda classification, and this can influence incidence estimates of cytological abnormalities. However, high rates of unsatisfactory anal cytology have been previously described in other cohorts, mainly attributed to inadequate sample collection [40]. Therefore, more standardization in sampling techniques is advisable to avoid misclassification.

## 5. Conclusions

A high prevalence of anal HPV infection and squamous intraepithelial lesions (SILs) was observed in our cohort of PLWH; furthermore, incidence estimates suggest a continuing increase over time in new infections and associated lesions.

This highlights the importance of strengthening immunization programs and continuous screening for anal HPV infection as well as for the presence of anal dysplasia in this population.

This study could not demonstrate the efficacy of the 9-valent HPV vaccine in preventing either the acquisition of HPV genotypes nor the appearance/evolution of pre-neoplastic lesions in PLWH (especially in patients already infected with another HPV genotype). However, a trend toward greater clearance of genotypes included in 9-valent HPV vaccine was observed in vaccinated patients, raising the question of whether HPV vaccine may be at least partially efficacious in stimulating the immune system of these patients. Given the small sample size and the still short duration of follow-up, considering the normal evolution of HPV infection, further prospective studies are needed to verify these results.

## Figures and Tables

**Table 1:** Population characteristics at baseline.

	N (%) or Median (IQR)
Age (years)	49 (43–56)
Male gender	94 (85.5)
Risk factor for HIV infection: Heterosexual Homosexual/bisexual IDU Other/unknown	16 (14.5) 60 (54.5) 1 (0.9) 33 (30)
Non Italian born	18 (16.4)
Years from HIV infection	8.5 (3.0–16.3)
CD4 at nadir (cells/mmc)	249 (107–420)
HIV-RNA at Zenith (log copies/mL)	4.98 (4.39–5.61)
Years from first ART	7 (3–13)
HBsAg positive	3 (2.7)
HBcAb positive	36 (32.7)
HCV Ab positive	5 (4.5)
History of syphilis	32 (29.1)
Past AIDS-related events	20 (18.2)
Type of ART: InSTI based PI based NNRTI based Dual Other	44 (40) 12 (10.9) 24 (21.8) 22 (20.0) 8 (7.3)
HIV-RNA < 50 copies/mL	93 (84.5)
CD4 (cells/mmc)	643 (459–873)
CD4:CD8 ratio	0.90 (0.60–1.30)
HPV Vaccination	34 (30.9)
HPV vaccination pre baseline	10/34 (9.1)

Abbreviations: ART, antiretroviral therapy; HCV, hepatitis C virus; IDU, injecting drug users; InSTI, integrase inhibitors; IQR, interquartile range; NNRTI, non-nucleoside reverse transcriptase inhibitors; PI, protease inhibitors.

**Table 2:** HPV infection and HPV-related cytological abnormalities at baseline and during follow-up.

	N (%) or Median (IQR)
HPV INFECTION at baseline	
Any HPV infection	95 (86.4)
HPV infection: No Low risk High risk	15 (13.6) 26 (23.6) 69 (62.7)
CYTOLOGY at baseline	
No abnormalities ASCUS LSIL HSIL Not evaluable	34 (30.9) 7 (6.4) 34 (30.9) 2 (1.8) 33 (30.0)
Evaluable follow-up	80 (72.7)
Follow-up, months	27.5 (0–45.5)
CYTOLOGY during follow-up (n = 80)	
Any abnormalities	57 (71.3)
HSIL	6 (7.5)
Worsening abnormalities	6 (7.5)
HPV INFECTION during follow-up	
HPV clearance (any genotype)	48 (60.0)
Low risk HPV genotype clearance	31 (38.8)
High risk HPV genotype clearance	30 (37.5)
New HPV infection	58 (72.5)
New Low Risk HPV infection	21 (26.3)
New High Risk HPV infection	49 (61.3)
New HPV infection from vaccinal genotype (in vaccinated)	13/21 (61.9)
New HPV infection from vaccinal genotype (in all)	39/73 (53.4)
Clearance of vaccinal HPV genotype (in vaccinated)	13/21 (61.9)
Clearance of vaccinal HPV genotype (in all)	39/73 (53.4)

Abbreviations: ASCUS, atypical squamous cells of uncertain significance; IQR, interquartile range; HSIL, high-grade squamous intraepithelial lesions; LSIL, low-grade squamous intraepithelial lesions.

**Table 3:** Comparison between HPV-vaccinated or unvaccinated patients.

	HPV-Vaccinated (*n* = 34)	HPV-Unvaccinated (*n* = 76)	*p*
Age (years)	47 (36–52)	50 (44–57)	0.014
Male gender	30 (88.2)	64 (84.2)	0.772
Risk factor: Heterosexual Homosexual/bisexual IDU Other/unknown	4 (11.8) 23 (67.6) 0 7 (20.6)	12 (15.8) 37 (48.7) 1 (1.3) 26 (34.2)	0.294
Non Italian born	5 (14.7)	13 (17.1)	1.000
Years from HIV infection	3.0 (2.0–13.5)	11.0 (5.0–17.0)	0.001
CD4 at nadir (cells/mmc)	261 (108–459)	242 (106–416)	0.969
HIV-RNA at Zenith (log copies/mL)	5.28 (4.26–5.65)	4.92 (4.42–5.54)	0.594
Years from first ART	3.0 (1.0–10.0)	8.0 (4.0–14.0)	0.001
HBsAg positive	0	3 (3.9)	0.551
HBcAb positive	7 (20.6)	29 (38.2)	0.081
HCV Ab positive	2 (5.9)	3 (3.9)	0.644
History of syphilis	9 (26.5)	23 (30.3)	0.821
Past AIDS-related events	5 (14.7)	15 (19.7)	0.603
Type of ART: InSTI-based PI-based NNRTI-based Dual other	19 (55.9) 4 (11.8) 6 (17.6) 3 (8.8) 2 (5.9)	25 (32.9) 8 (10.5) 18 (23.7) 19 (25.0) 6 (7.9)	0.151
HIV-RNA < 50 copies/mL	29 (85.3)	64 (84.2)	1.000
CD4 (cells/mmc)	630 (394–900)	648 (472–865)	0.527
CD4:CD8 ratio	0.90 (0.50–1.49)	0.90 (0.60–1.30)	0.895

Abbreviations: ART, antiretroviral therapy; HCV, hepatitis C virus; IDU, injecting drug users; InSTI, integrase inhibitors; IQR, interquartile range; NNRTI, non-nucleoside reverse transcriptase inhibitors; PI, protease inhibitors.

**Table 4:** HPV infection and HPV-related cytological abnormalities at baseline and during follow-up in unvaccinated versus vaccinated patients.

	HPV Vaccinated (*n* = 34)	HPV Unvaccinated (*n* = 76)	*p*
HPV INFECTION at baseline			
Any HPV infection	28 (82.4)	67 (88.2)	0.604
HPV infection: -No -Low risk genotypes -High risk genotypes	6 (17.6) 9 (26.5) 19 (55.9)	9 (11.8) 17 (22.4) 50 (65.8)	0.573
CYTOLOGY at baseline			
-No abnormalities -ASCUS -LSIL -HSIL -Not evaluable	11 (32.4) 0 12 (35.3) 0 11 (32.4)	23 (30.3) 7 (9.2) 22 (28.9) 2 (2.6) 22 (28.9)	0.348
Evaluable follow-up	28 (82.4)	52 (68.4)	0.199
Follow-up, months	36.9 (16.6–47.9)	20.0 (0–45.2)	0.036
CYTOLOGY during Follow-up			
Any abnormalities	20 (58.8)	45 (59.2)	1.000
HSIL	2 (5.9)	4 (5.3)	1.000
Worsening abnormalities	3 (8.8)	3 (3.9)	0.371
HPV INFECTION during Follow-up			
HPV clearance	18 (52.9)	30 (39.5)	0.268
Low risk HPV genotype clearance	13 (38.2)	18 (23.7)	0.181
High risk HPV genotype clearance	11 (32.4)	19 (25.0)	0.570
New HPV infection	24 (70.6)	34 (44.7)	0.021
New Low Risk HPV infection	9 (26.5)	12 (15.8)	0.292
New High Risk HPV infection	20 (58.8)	29 (38.2)	0.071
New HPV infection from vaccinal Genotype (in all)	13/21 (61.9)	26/74 (35.1)	0.051
Clearance of vaccinal HPV (in all)	13/21 (61.9)	26/74 (35.1)	0.051

Abbreviations: ASCUS, atypical squamous cells of uncertain significance; IQR, interquartile range; HSIL, high-grade squamous intraepithelial lesions; LSIL, low-grade squamous intraepithelial lesions.

**Table 5:** Variables associated with the occurrence of a new HPV infection or clearance of any HPV genotype (Cox regression analysis).

	Univariate Analysis		Multivariate Analysis	
	HR (95% CI)	*p*	aHR (95% CI)	*p*
Predictors of any new HPV genotype infection:				
AIDS	0.40 (0.17–0.95)	0.037	0.48 (0.20–1.16)	0.103
CD4 > 500 cells/mmc	1.89 (1.03–3.47)	0.041	1.58 (0.84–2.97)	0.155
Predictors of clearance of any HPV genotype:				
HR-HPV versus LR-HPV genotypes	0.50 (0.28–0.91)	0.023	0.56 (0.31–1.01)	0.054
CD4 > 500 cells/mmc	2.62 (1.24–5.54)	0.011	2.44 (1.15–5.18)	0.021

Abbreviations: CI; confidence intervals; aHR, adjusted hazard ratio; HR, hazard ratio; HR-HPV, high risk HPV genotypes; LR-HPV, low risk HPV genotypes.

## Data Availability

The data presented in this study are available on request from the corresponding author due to privacy and ethical reasons.

## Supplementary Materials

The following supporting information can be downloaded at: https://www.mdpi.com/article/10.3390/diagnostics15020198/s1, Figure S1: Kaplan–Meier estimates of the Incidence of HPV infection/clearance and evolution of cytological abnormalities over time.
