# Supplementary material for: Prevalence, Incidence and Predictors of Anal HPV Infection and HPV-Related Squamous Intraepithelial Lesions in a Cohort of People Living with HIV
Source: Diagnostics (Basel). 2025 Jan 16;15(2):198. doi: 10.3390/diagnostics15020198 (PMC11763758; doi:10.3390/diagnostics15020198)
Supplement: Supplementary file 1 [file diagnostics-15-00198-s001.zip › diagnostics-3390608-supplementary.pdf]

Supplementary Figure S1: Kaplan-Meier estimates of the Incidence of HPV infection/clearance and evolution of cytological abnormalities over time

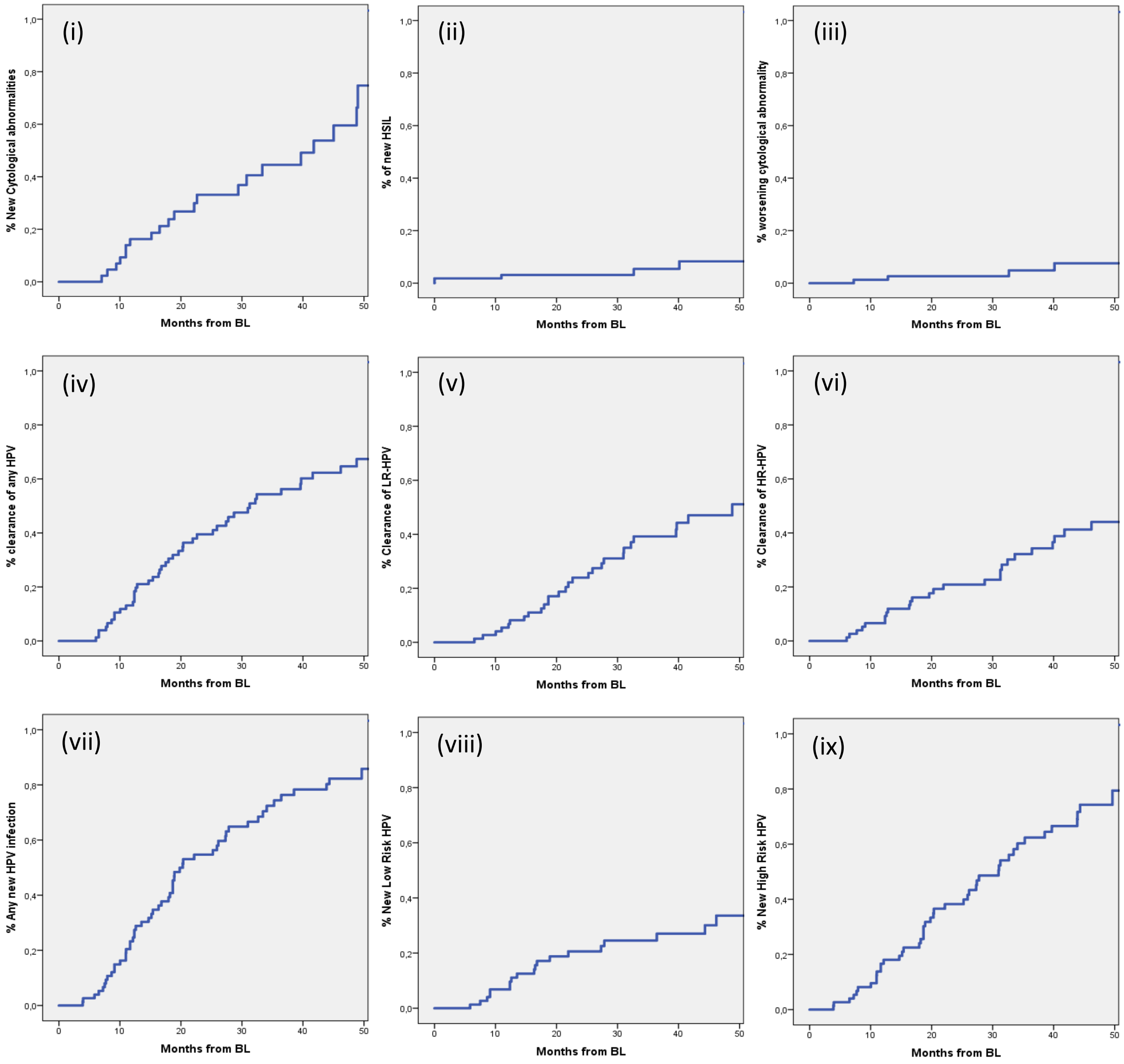

LEGEND

(i) any new cytological abnormality
(ii) new HSIL
(iii) worsening cytological abnormalities
(iv) clearance of any HPV
(v) clearance of LR-HPV
(vi) clearance of HR-HPV
(vii) any new HPV infection
(viii) new LR-HPV infection
(ix) new HR-HPV infection
